# Influence of the strain effect on magnetocrystalline anisotropy in Co_2_Fe_0.4_Mn_0.6_Si Heusler alloys

**DOI:** 10.1038/s41598-023-43979-x

**Published:** 2023-10-09

**Authors:** A. Nabiałek, O. M. Chumak, P. Aleshkevych, J. Z. Domagala, A. Pacewicz, B. Salski, J. Krupka, T. Seki, K. Takanashi, L. T. Baczewski, H. Szymczak

**Affiliations:** 1 Institute of Physics, Polish Academy of Sciences, 02-668 Warsaw, Poland; 2 Institute of Radioelectronics and Multimedia Technology, Warsaw University of Technology, 00-665 Warsaw, Poland; 3 Institute for Materials Research, Tohoku University, Sendai, 980-8577 Japan; 4 Advanced Science Research Center, Japan Atomic Energy Agency, Tokai, 319-1195 Japan

**Keywords:** Nanoscale materials, Magnetic properties and materials, Surfaces, interfaces and thin films

## Abstract

The perpendicular magnetocrystalline anisotropy, magnetoelastic properties as well as the Gilbert damping factor in Co_2_Fe_0.4_Mn_0.6_Si thin films were found to depend on a magnetic layer thickness, and they can be also tuned by the application of additional Ag buffer layer. The tetragonal distortion of a magnetic layer was found to increase with decreasing thickness, and after the application of an additional Ag buffer layer, the character of this distortion was changed from tensile to compressive in the plane of a film. A correlation between the tetragonal distortion and perpendicular magnetocrystalline anisotropy was found. However, the magnitude of the observed tetragonal distortion for most samples seems to be too small to explain alone the experimentally found large magnitude of the perpendicular magnetocrystalline anisotropy. For these samples, other mechanisms including both surface and volume effects must be taken into account.

## Introduction

Thanks to a high spin polarization, Co-based Heusler alloys are promising materials for applications in spintronics^1–3^. In such applications heterostructures consisting of several magnetic or nonmagnetic conducting layers are used. If the thickness of the magnetic layer is sufficiently small, its properties differ from those of bulk material because of the surface effects, which is inversely proportional to the layer thickness. Among a large number of surface effects, in the case of magnetic layers, magnetic surface anisotropy^4^ is especially important.

When the layers thickness is of an order of nanometers, the presence of surface anisotropy may change an easy axis of magnetization from parallel to perpendicular to the layer surface. Such a perpendicular magnetic anisotropy (PMA) is often observed in 1–2 nm thick magnetic layers of cobalt^5,6^. PMA was also observed in 0.6–0.8 nm thick Co_2_Fe_x_Mn_1−x_Si films with a palladium buffer layer^7^. For the Heusler alloy layers with larger thicknesses, PMA is not expected. Nevertheless, controlling the magnetic anisotropy is important because of its possible correlation with coercivity, and thus with dissipation processes. The magnetic anisotropy plays an important role when creating, predicted by the theory^8^, topologically protected nontrivial spin textures including magnetic skyrmions or bimerones^9,10^. Such topological quasiparticles with nanoscale size and high mobility have potential applications in information storage and spintronic devices^11^. Magnetic skyrmions were also observed in heterostructures containing ultrathin Co-based Co_2_FeAl (CFA) Heusler alloy^12,13^. The topology and creation of magnetic skyrmions in Heusler compounds were recently discussed in a review paper^14^.

If magnetoelastic effects are present in the magnetic layer, magnetic anisotropy can be also induced by the strain. Recently, the strain effect was shown to be a prime source of perpendicular magnetic anisotropy presence in Ni/Pt epitaxial superlattice^15^. The tetragonal distortion of the magnetic layer was also shown, both experimentally and by the first principles electronic structure calculations, to correlate with the perpendicular magnetocrystalline anisotropy in the Mn_2−δ_CoGa_1+δ_ thin films^16^. The hybridization between two states with opposite spins split by the crystal field was proposed as the cause of magnetocrystalline anisotropy for these materials^16^. The strain-induced magnetic anisotropy for one Co_2_Fe_0.4_Mn_0.6_Si (CFMS) sample was also estimated in our previous paper^17^. The estimated magnitude of the strain induced magnetocrystalline anisotropy seemed to be too small to explain large perpendicular magnetocrystalline anisotropy constant. However, in order to analyze a possible correlation between the tetragonal distortion and the anisotropy, the comparison of the samples characterized by different distortions is necessary.

The magnetoelastic properties are also influenced by surface effects, which can be expressed in terms of so-called “surface magnetostriction” or “surface magnetoelastic coupling”^18,19^. Hence, in the case of thin films, the adoption of the magnetoelastic constants of bulk materials may lead to significant errors. The first principles calculations for the Co_2_XAl (X=V, Ti, Cr, Mn, Fe) full Heusler compounds show that the magnetoelastic properties of these compounds may be also anisotropic^20^.

In our recent work^21^ the magnetoelastic properties of a series of 30 nm thick Co_2_Fe_x_Mn_1−x_Si films with different Fe contents were investigated. Magnetoelastic properties were found to be anisotropic in the plane of the film and to change with the sample composition. The magnetoelastic properties, cubic magnetocrystalline anisotropy constant as well as Gilbert damping factor were found to be correlated with the band structure of the studied materials. The samples revealed also perpendicular magnetocrystalline anisotropy constant with a surprisingly large magnitude (of an order 10^6^ erg/cm^3^).

In this work, we present a comparison of the magnetoelastic properties in CFMS thin films of different thicknesses (15 nm, 30 nm and 50 nm). Additionally, an effect of an additional Ag buffer layer application is studied. In particular, the magnetocrystalline anisotropy and anisotropic magnetoelastic properties were investigated. Determination of the lattice constants, using the x-ray studies, enabled also the calculation of the component of the magnetocrystalline anisotropy induced by a strain, and comparison with the total perpendicular magnetocrystalline anisotropy determined experimentally using the ferromagnetic resonance (FMR) technique.

Finally, a possible correlation between magnetoelastic properties and magnetic damping at microwave frequencies is also discussed.

## Experimental details

The ultrahigh-vacuum-compatible magnetron sputter-deposition was employed to obtain the epitaxially grown CFMS thin films. The magnetic layers with the thickness of 15 nm (sample 15 nm), 30 nm (sample 30 nm) or 50 nm (sample 50 nm) were deposited on the 0.5 mm thick MgO (100) substrate with the 20 nm Cr buffer layer to obtain a low surface roughness. Other samples with the 30 nm and 50 nm thick CFMS layer were grown on an additional 20 nm Ag buffer layer deposited on the Cr layer (samples 30 nm/Ag and 50 nm/Ag, respectively). All the samples were covered by a 5 nm Au capping layer to prevent oxidation. The in-situ reflection high-energy electron diffraction (RHEED) and ex-situ X-ray diffraction studies showed the CFMS layers to be epitaxial with the cubic symmetry and with high degree (~ 80%) *B*2 and moderate (~ 20%) *L*2_1_ orderings.

To determine a possible tetragonal distortion of the cubic structure, additional investigations were performed using a high-resolution X-ray diffractometer Panalytical Empyrean with a hybrid two-bounce Ge (220) monochromator, with radiation of Cu_Kα1_ (1.5406 Å) and a 2D detector PIXcel.

The magnetoelastic properties of the films were determined using the strain-modulated FMR (SMFMR) technique^22^. In this technique, the shift of the FMR resonance line caused by a periodic (frequency of about 48 kHz) strain is measured. The SMFMR experiments were performed in two orientations of a sample with the external in-plane magnetic field applied parallel to the [100] or [110] crystallographic axis of the CFMS layer. Such geometry of the experiment enabled the determination of two magnetoelastic constants characteristic for crystals with cubic symmetry.

To determine the perpendicular magnetocrystalline anisotropy constant, additional FMR experiments in an external out-of-plane magnetic field (up to 1.8 Tesla) were performed. To increase the accuracy of the anisotropy constants determination the whole angular dependences of the FMR resonance field were registered. The saturation magnetization of the magnetic layers was determined using a vibrating sample magnetometer (VSM).

The Gilbert damping factor was determined using the vector network analyzer (VNA). In our VNA experiments, the film was mounted on a micro-strip line, and the frequency of the microwaves was varied in the range from 4 to 20 GHz. The external magnetic field was applied in plane of the film and parallel to the [100] axis of the CFMS layer.

## Results

### Tetragonal distortion

Table 1 presents the lattice constants of the CFMS magnetic layers of the five films studied in the experiments. The lattice constants were measured in the directions both parallel and perpendicular to the film plane. The difference between both constants reveals a presence of the tetragonal distortion in the studied samples.

**Table 1:** The in-plane and perpendicular-to-the-film-plane lattice constants of the 15 nm, 30 nm and 50 nm (with and without Ag layer) CFMS layers.

Layer thickness	Lattice constant in plane (Å)	Lattice constant perpendicular (Å)	*ε*_11_ = *ε*_22_	*ε*_33_
15 nm	5.6878	5.6258	5.1 × 10^–3^	− 5.9 × 10^–3^
30 nm	5.6696	5.6503	1.6 × 10^–3^	− 1.8 × 10^–3^
50 nm	5.6610	5.6555	4.5 × 10^–4^	− 5.2 × 10^–4^
30 nm/Ag	5.6457	5.6685	− 1.9 × 10^–3^	2.2 × 10^–3^
50 nm/Ag	5.6450	5.6750	− 2.5 × 10^–3^	2.9 × 10^–3^

The calculated tetragonal (ε_11_ = ε_22_ and ε_33_) distortions are also shown.

The in-plane (*a*_*in*_) and perpendicular-to-plane (*a*_per_) lattice constants can be calculated according to the formulas:

1 $$a_{in} = a_{0} + a_{0} \varepsilon_{11}$$

2 $$a_{per} = a_{0} + a_{0} \varepsilon_{33} ,$$

where *a*_0_ is the lattice constant of the relaxed (unstrained) magnetic layer and *ε*_11_ = *ε*_22_ and *ε*_33_ are the strains of the magnetic layer in-plane and perpendicular to the plane, respectively. Additionally

3 $$\varepsilon_{33} = - 2\frac{{c_{12} }}{{c_{11} }}\varepsilon_{11} ,$$

where *c*_11_ and *c*_12_ are the elastic constants. Solving Eqs. (1)–(3), we got the strain values presented in Table 1. We assumed *c*_11_ = 296 GPa and *c*_12_ = 172 GPa (interpolated data for the Co_2_FeSi and Co_2_MnSi compounds taken from Ref.^23^). It can be seen from Table 1 that the application of an additional Ag buffer layer changes the distortion in the plane of the film from tensile to compressive. In the case of the samples without the Ag buffer layer, a decrease in the magnetic layer thickness from 50 to 15 nm leads to an increase of the tetragonal distortion by one order of magnitude. The lattice constant of the relaxed structure for all the samples was found to be *a*_0_ = 5.659 ± 0.003 Å.

### Magnetoelastic properties

**Figure 1:**
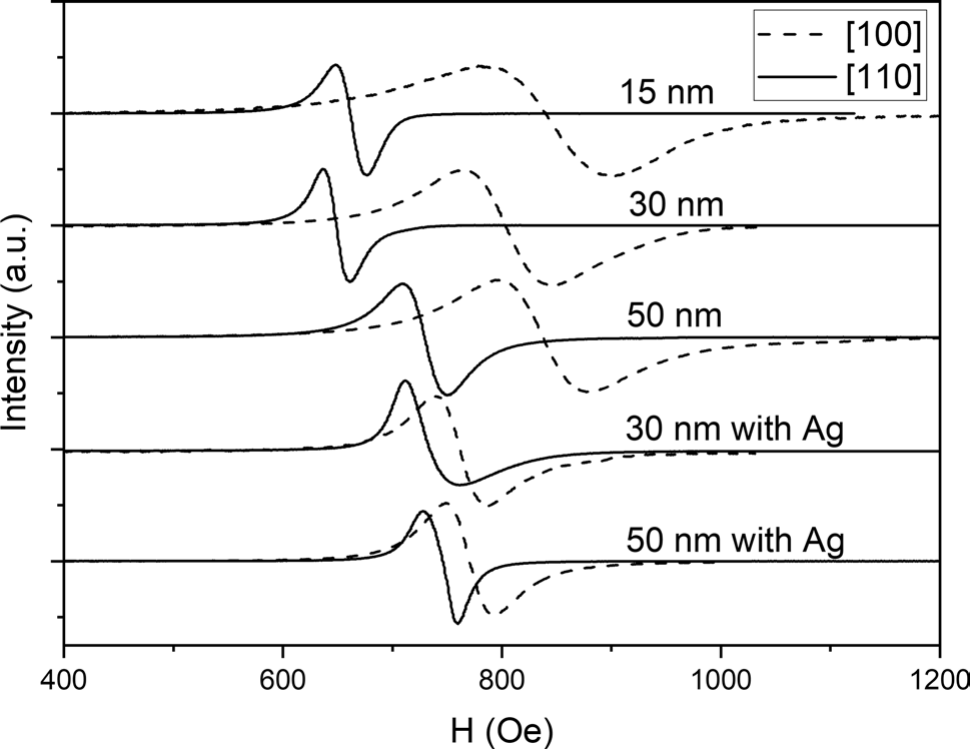
The SMFMR curves with normalized amplitudes of the five samples studied in our experiments. The external magnetic field was applied along the in-plane [100] and [110] crystallographic axis of the epitaxial film.

The SMFMR curves with normalized amplitudes of the five samples studied in our experiments are shown in Fig. 1.

Figure 1 shows that changing the thickness of the CFMS layer, as well as the application of the Ag buffer layer definitely changes the in-plane magnetocrystalline anisotropy, which manifests itself by changing the resonant fields measured along the [100] and [110] axes. More detailed studies of magnetocrystalline anisotropy are presented in the next section.

To determine the magnetoelastic constants of the investigated magnetic layers, we used the same procedure as that described in detail in our previous work^21^. By comparing the amplitudes of two FMR curves, one of which is modulated by the magnetic field (with known amplitude) and the other one—by the strain, we could determine the shift of the FMR line caused by the strain, which are given in Table 2.

**Table 2:** The FMR line shifts for the five samples studied in our experiments measured in-plane of the film along the [100] (∆H_100_) and [110] (∆H_110_) axes of the epitaxial magnetic layer, caused by a periodic strain of the quartz rod to which the samples were glued.

Sample	∆H_100_ (Oe)	ε_11_^*^ (10^–6^)	ε_22_^*^ (10^–5^)	∆H_110_ (Oe)	ε_11_^*^ (10^–6^)	ε_22_^*^ (10^–5^)
15 nm	0.50	− 3.1	1.78	1.03	− 3.1	1.54
30 nm	0.70	− 3.0	1.58	0.92	− 2.7	1.40
50 nm	0.73	− 2.5	1.41	0.89	− 2.5	1.31
30 nm/Ag	0.67	− 2.7	1.67	1.10	− 3.0	1.56
50 nm/Ag	0.57	− 2.8	1.61	0.90	− 3.2	1.66

ε_11_* and ε_22_* denote the amplitudes of the periodic strain in the directions perpendicular and parallel to the rod, respectively.

The shifts of the FMR lines shown in Table 2 were next used to calculate magnetoelastic constants. The magnetoelastic energy can be described by the formula:

4 $$E_{me} = b_{1} \left( {\alpha_{1}^{2} \varepsilon_{11} + \alpha_{2}^{2} \varepsilon_{22} + \alpha_{3}^{2} \varepsilon_{33} } \right) + 2b_{2} \left( {\alpha_{1} \alpha_{2} \varepsilon_{12} + \alpha_{2} \alpha_{3} \varepsilon_{23} + \alpha_{1} \alpha_{3} \varepsilon_{13} } \right){ },$$

where *b*_1_ and *b*_2_ are the two cubic magnetoelastic constants. α_i_ are direction cosines, and *ε*_ij_ are the components of the strain tensor expressed in the coordinate system associated with the sample. The calculated magnetoelastic constants are shown in Table 3.

**Table 3:** The calculated cubic magnetoelastic constants *b*_1_ and *b*_2_ of the 15 nm, 30 nm and 50 nm CFMS layers (with and without Ag buffer).

Sample	*b*_1_ (erg/cm^3^)	*b*_2_ (erg/cm^3^)
15 nm	− 1.24 × 10^7^	− 2.84 × 10^7^
30 nm	− 1.91 × 10^7^	− 2.79 × 10^7^
50 nm	− 2.28 × 10^7^	− 2.94 × 10^7^
30 nm/Ag	− 1.71 × 10^7^	− 2.90 × 10^7^
50 nm/Ag	− 1.68 × 10^7^	− 2.49 × 10^7^

Similarly to the samples studied in Ref.^21^, the magnetoelastic properties are anisotropic, i.e. the *b*_1_ and *b*_2_ constants for each sample have different magnitudes (for an isotropic sample, *b*_1_ = *b*_2_), and the magnitude of *b*_2_ constant is always higher than *b*_1_. Additionally, the magnetoelastic constants are also changing with the thickness of the magnetic layer, and their magnitudes can be tuned by the application of an additional Ag buffer layer.

The saturation magnetization of the magnetic layers measured by VSM was 979 emu/cm^3^, 967 emu/cm^3^, 972 emu/cm^3^, 930 emu/cm^3^, and 1040 emu/cm^3^ for the 15 nm, 30 nm, 50 nm, 30 nm/Ag and 50 nm/Ag samples, respectively. A slightly higher magnitude of saturation magnetization for the 50 nm/Ag sample may suggest some improvement of the chemical ordering for the thickest sample with an additional Ag buffer layer.

### Magnetocrystalline anisotropy

In the SMFMR measurement system, there is no possibility to rotate the external magnetic field in the plane of the film. To take each FMR curve shown in Fig. 1 the sample must be re-glued. Hence, taking this way an accurate angular dependence of the resonant field is very difficult. Additionally, the maximal external magnetic field attainable in our SMFMR system was only about 9500 Oe, which make the determination of the resonant field perpendicular to the plane of the film, and the perpendicular magnetocrystalline anisotropy, impossible. For this reason, additional FMR experiments were performed using Bruker EMX EPR spectrometer.

**Figure 2:**
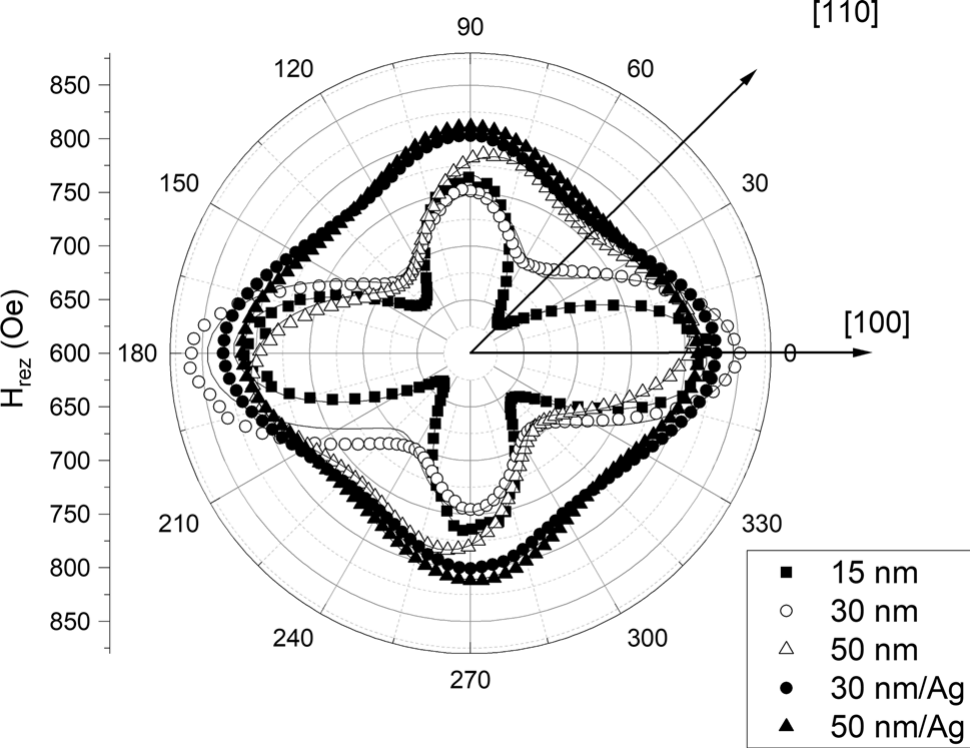
In-plane angular dependences of the FMR resonance fields for the five samples studied in our experiments. Solid lines represent the fitting curves.

The angular dependences of the in-plane FMR resonant field for the three samples studied in our experiments are shown in Fig. 2. The four-fold symmetry of the curves confirms that in the plane of the film the cubic magnetocrystalline anisotropy is dominant. It can be also seen that increasing the thickness of the CFMS layer as well as adding the Ag buffer layer decreases this anisotropy (i.e. the curves become more circular). For the samples without the Ag buffer layer, as well for the thinner 30 nm sample with Ag buffer, the dominant four-fold symmetry is slightly broken suggesting the presence of the component of the in-plane uniaxial symmetry.

**Figure 3:**
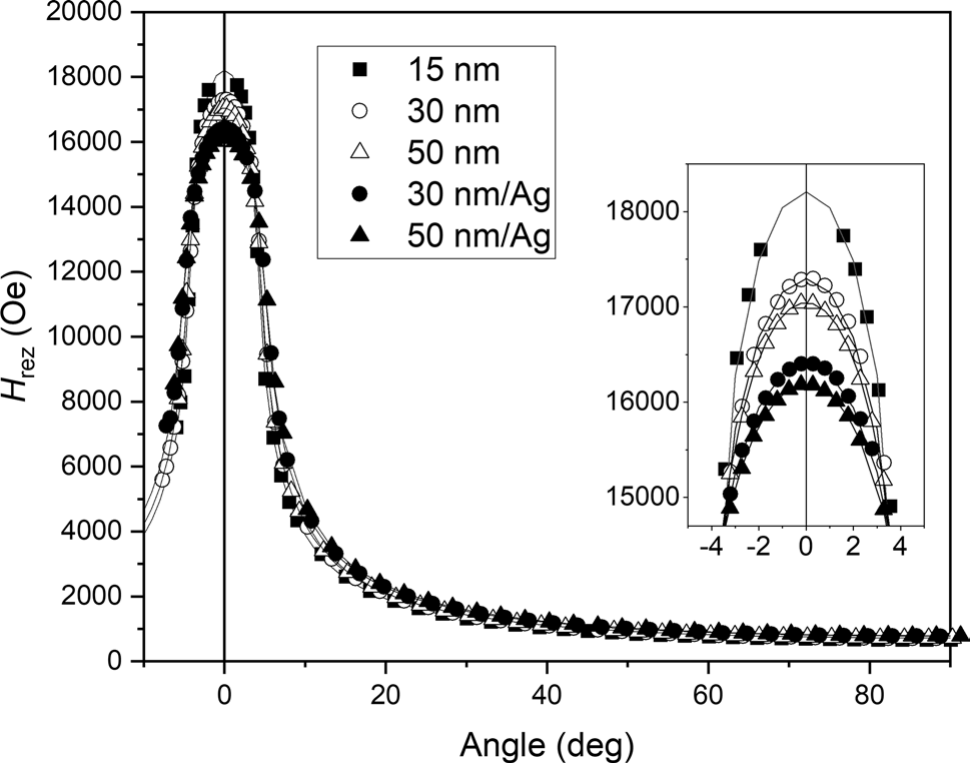
Out-of-plane angular dependences of the resonance field for the five samples studied in our experiments. Solid lines represent the fitting curves. The zero angle corresponds to the perpendicular direction to the film plane. The inset presents magnified plot near zero angle.

The out-of-plane angular dependences of the FMR resonance field for the five samples studied in our experiments are shown in Fig. 3.

The zero angle in Fig. 3 corresponds to the orientation of the external magnetic field perpendicular to the film and 90 degree corresponds to the field in-plane of the film and parallel the [110] axis of the epitaxial CFMS magnetic layer. One can see that increasing the thickness of the magnetic layer and adding the Ag buffer layer decrease the magnitude of the perpendicular resonant magnetic field, suggesting a decrease in perpendicular magnetic anisotropy.

To fit the experimental data (shown in Figs. 2 and 3), we used the formula for the free energy of the sample:

5 $$F = - \mathop \sum \limits_{i = 1}^{3} M_{i} H_{i} + 2\pi M_{s}^{2} \alpha_{3}^{2} - K_{p} \alpha_{3}^{2} + K_{p2} \alpha_{3}^{4} + K_{1} \left( {\alpha_{1}^{2} \alpha_{2}^{2} + \alpha_{2}^{2} \alpha_{3}^{2} + \alpha_{1}^{2} \alpha_{3}^{2} } \right) - K_{U} \left( {n_{1}^{2} \alpha_{1}^{2} + n_{2}^{2} \alpha_{2}^{2} } \right)$$

The first two terms in Eq. (5) are the Zeeman and magnetostatic energies. *M*_s_ is the saturation magnetization and *H* is the external magnetic field. α_i_ denotes the direction cosines of the magnetization vector in the coordinate system associated with the sample. The third coordinate is perpendicular to the film, and the first one is parallel to the [100] axis of the epitaxial CFMS layer. We assumed that the out-of-plane magnetocrystalline anisotropy can be described by the two terms proportional to ${\alpha }_{3}^{2}$ and ${\alpha }_{3}^{4}$, respectively [see the third and fourth term in Eq. (5)]. The fifth term in Eq. (5) is the cubic magnetocrystalline anisotropy with the first cubic magnetocrystalline constant ${K}_{1}$. The sixth term in Eq. (5) describes the in-plane component of the uniaxial anisotropy, whose magnetization easy axis is defined by a unit vector ${\mathrm{n}}_{\mathrm{i}}$.

The FMR resonance conditions can be calculated using the set of equations^24^:

6 $$\left( {\frac{\omega }{\gamma }} \right)^{2} = \frac{1}{{M_{s}^{2} \sin^{2} \theta }}\left[ {\left( {\frac{{\partial^{2} F}}{{\partial \theta^{2} }}} \right)\left( {\frac{{\partial^{2} F}}{{\partial \varphi^{2} }}} \right) - \left( {\frac{{\partial^{2} F}}{\partial \theta \partial \varphi }} \right)^{2} } \right]$$

and

7 $$\frac{\partial F}{{\partial \theta }} = \frac{\partial F}{{\partial \varphi }} = 0 ,$$

where *ω* is the angular frequency, *γ* the gyromagnetic ratio, *θ* and *φ* are coordinates of a polar system ($\alpha_{1} = \cos \varphi \sin \theta$, $\alpha_{2} = \sin \varphi \sin \theta$, $\alpha_{3} = \cos \theta$).

Our fitting procedure has taken into account both in-plane and out-of-plane angular resonance field dependences, and the fitting curves are presented as solid lines in Figs. 2 and 3. The fitting parameters are shown in Table 4.

**Table 4:** The magnetocrystalline anisotropy constants [defined by Eq. (5)] obtained from the fitting procedure for the five samples studied in the experiments.

Sample	15 nm	30 nm	50 nm	30 nm/Ag	50 nm/Ag
*K*_p_ (erg/cm^3^)	− 1.0 × 10^6^	− 6.2 × 10^5^	− 5.3 × 10^5^	− 6.2 × 10^5^	1.0 × 10^5^
*K*_p2_ (erg/cm^3^)	1.2 × 10^5^	1.3 × 10^5^	1.1 × 10^5^	1.1 × 10^4^	~ 0
*K*_1_ (erg/cm^3^)	− 3.7 × 10^4^	− 2.5 × 10^4^	− 1.7 × 10^4^	− 1.1 × 10^4^	− 1.1 × 10^4^
*K*_U_ (erg/cm^3^)	1.4 × 10^4^	2.4 × 10^4^	1.7 × 10^4^	6.5 × 10^3^	~ 0
_ϕU_ (deg)	79	92	126	85	–
g	2.034	2.057	2.056	2.024	2.035

The angle ϕ_U_ defines the deviation of an easy axis of the in-plane component of the uniaxial anisotropy from the [100] direction of the epitaxial CFMS layer. The values of the gyromagnetic factor, g, obtained from the fitting procedure are also shown.

It can be seen that increasing the thickness of the CFMS layer decreases the magnitude of the perpendicular anisotropy constant, *K*_p_ and moreover for the 50 nm sample with an additional Ag buffer layer, *K*_p_ changes its sign. The negative value of *K*_p_ means that the in-plane magnetization orientation is preferred, while the positive value prefers the perpendicular magnetization orientation. However, the magnitude of *K*_p_ = 1.0 × 10^5^ erg/cm^3^ is too small to overcome the demagnetizing energy (about 6.8 × 10^6^ erg/cm^3^ for the sample with the Ag buffer layer). To describe the angular dependence of the resonance field of the 50 nm sample with the Ag buffer layer it is sufficient to consider only one perpendicular anisotropy constant *K*_p_. In the case of the samples without the Ag layer, as well as the 30 nm/Ag sample, for better fit, it is necessary to consider also the second perpendicular anisotropy constant *K*_p2_.

For all investigated samples the first cubic anisotropy constant is negative, which means that the in-plane easy axis of magnetization is parallel to [110] axis of the epitaxial CFMS layer. However, the magnitude of this constant decreases with increasing thickness of the CFMS layer, and an additional decrease in its magnitude was found after the application of the Ag buffer layer. To describe the in-plane angular dependence of the resonance field of the samples without the Ag layer, as well as the 30 nm/Ag sample, it was also necessary to take into account an additional component of the uniaxial anisotropy, *K*_u_, lying in the plane of the film. The direction of this anisotropy axis coincides neither with the [100] nor [110] crystallographic axis of the CFMS layer. However, for the 15 nm, 30 nm and 30 nm/Ag samples the direction of this anisotropy axis is closer to [010] and for the 50 nm sample closer to [− 110] crystallographic axis of the CFMS layer. The angle ϕ_U_ which defines the deviation of an easy axis of the in-plane component of the uniaxial anisotropy from the [100] direction of the epitaxial CFMS layer is given in Table 3.

## Discussion

Comparing the experimental data of the lattice tetragonal distortion (Table 1) and the perpendicular magnetocrystalline anisotropy (Table 4), one can find a qualitative correlation between both parameters. For the samples without Ag buffer, the magnitudes of both parameters decrease with increasing thickness of the CFMS layer. For the 50 nm/Ag sample both the tetragonal distortion and *K*_p_ change their signs.

The knowledge of both the magnetoelastic constants and the tetragonal distortion of the CFMS layers enabled a calculation of the magnetocrystalline anisotropy component induced by the strain. In the case of tetragonal distortion (ε_11_ = ε_22_, ε_12_ = ε_23_ = ε_13_ = 0) the magnetoelastic energy [Eq. (4)] can be expressed by the formula:

8 $$E_{mc} = - b_{1} \left( {\varepsilon_{11} - \varepsilon_{33} } \right)\alpha_{3}^{2} = - K_{si} \alpha_{3}^{2} ,$$

where $K_{si} = b_{1} \left( {\varepsilon_{11} - \varepsilon_{33} } \right)$ is the strain-induced perpendicular anisotropy. Table 5 shows a comparison between the calculated *K*_si_ and the perpendicular magnetocrystalline anisotropy *K*_p_ determined from FMR experiments (taken from Table 4).

**Table 5:** Comparison between the perpendicular magnetocrystalline anisotropy *K*_p_ and the strain-induced perpendicular anisotropy *K*_si_ for the studied samples.

Sample	*K*_p_ (erg/cm^3^)	*K*_si_ (erg/cm^3^)
15 nm	− 1.0 × 10^6^	− 1.4 × 10^5^
30 nm	− 6.2 × 10^5^	− 0.6 × 10^5^
50 nm	− 5.3 × 10^5^	− 0.2 × 10^5^
30 nm/Ag	− 6.2 × 10^5^	0.7 × 10^5^
50 nm/Ag	1.0 × 10^5^	0.9 × 10^5^

For the samples without Ag buffer both *K*_p_ and *K*_si_ are negative, and for the 50 nm/Ag sample positive. The magnitudes of *K*_si_ for most samples (except for 50 nm/Ag), are at least several time smaller than *K*_p_. The magnitudes of *K*_p_ decrease with increasing thickness of magnetic layer. The lower magnitudes of *K*_si_ (in comparison with *K*_p_) suggest that the strain-induced magnetocrystalline anisotropy is insufficient to explain such large perpendicular magnetocrystalline anisotropy.

For this reason, the most probable dominant mechanism responsible for increasing the magnitude of *K*_p_ with decreasing thickness of the CFMS layer in these samples seems to be the surface anisotropy^4^. It should be noted that also in the case of the surface anisotropy mechanism the magnitude of *K*_p_ is expected to increase with decreasing thickness of the magnetic layer. We assumed:

9 $$K_{p} \left( d \right) = K_{V} + \frac{{K_{S} }}{d} + K_{si} \left( d \right),$$

where *d* is the thickness of CFMS layer. *K*_V_ and *K*_S_ are the volume and surface components of the perpendicular magnetocrystalline anisotropy, respectively. *K*_S_ consists of two components connected with two interfaces of the magnetic layer, and it is expected to be different for the samples with and without the Ag buffer layer. Fitting the results according to Eq. (9) gives *K*_V_ = − 3.3 × 10^5^ erg/cm^3^ and *K*_S_ = − 0.78 erg/cm^2^ for the samples without Ag buffer, and *K*_V_ = 1.0 × 10^6^ erg/cm^3^ and *K*_S_ = − 5.25 erg/cm^2^ for the samples with Ag buffer layer, which shows that the contributions of both *K*_V_ and *K*_S_ for both series of the samples are different.

It should be noted that, according to our FMR data, the anisotropy of the CFMS layers deposited without an additional Ag buffer layer, as well as the 30 nm/Ag sample, is more complex than that of the cubic crystal with tetragonal distortion. In particular, the presence of the in-plane component of the uniaxial anisotropy should be taken into account. In our analysis, we assumed that the magnetoelastic properties of the magnetic layer can be described only by the two magnetoelastic constants characteristic for the cubic crystals, which may lead to significant errors.

We already studied the influence of the CFMS layer thickness on their magnetoelastic properties in Ref.^17^. However, we assumed that the in-plane magnetoelastic properties are isotropic. In Ref.^21^, we showed that the anisotropic in-plane properties change with the magnetic layer composition, showing also a correlation with the band structure of the investigated materials. Present results show that both the magnetocrystalline anisotropy and the two cubic magnetoelastic constants of the CFMS layers change with the thickness of the magnetic layer, and they are also dependent on the type of buffer layer on which the CFMS layer is deposited.

In the paper^17^, the changes in the Gilbert damping factor with the thickness of the magnetic layer were also studied. It was found that for the samples for which the spin pumping phenomenon^25^ can be neglected the Gilbert damping factor also increases with increasing thickness of the magnetic layer suggesting a correlation with magnetoelastic properties.

Both magnetoelastic properties and magnetocrystalline anisotropy can influence the magnetic damping. However, in the case of conducting magnetic layers, like CFMS, also other phenomena, including spin pumping^25^ and eddy currents^26^, should be taken into account. The spin pumping phenomenon leads (unlike in our samples) to an increase of Gilbert damping with decreasing thickness of the magnetic layer. In fact, just the opposite effect (i.e. an increase of damping factor with increasing thickness of the magnetic layer) is expected to be induced by the eddy currents^26^.

For the lowest mode in FMR, the contribution of the eddy currents to the Gilbert damping factor for the sample with thickness δ and resistivity ρ can be expressed by the formula^26^:

10 $$\alpha^{eddy} = \frac{C}{16}\frac{{\gamma \mu_{0}^{2} M_{s} \delta^{2} }}{\rho }$$

In Eq. (10), μ_0_ is vacuum permeability, and *C* is a phenomenological parameter depending on the eddy current distribution in the ferromagnet (0 ≤ C ≤ 1) ^26^. Let us assume maximal value of *C* = 1 and the parameters characteristic for our samples i.e. ρ ≈ 173 μΩ cm^27^ and *M*_s_ ~ 1000 emu/cm^3^. For the 15 nm and 50 nm, we obtain ${\mathrm{\alpha }}^{\mathrm{eddy}}$ to be about 2.3 × 10^–6^ and 2.5 × 10^–5^, respectively. On the other hand, the Gilbert damping factor estimated from our broadband FMR studies for the CFMS films changes from about 1 × 10^–3^ for the 15 nm sample to about 4.5 × 10^–3^ sample for the 50 nm sample^17^. These values are at least two orders of magnitude higher than the Gilbert damping factor calculated from the eddy current model, suggesting that for the CFMS films with the thickness lower than 50 nm the influence of the eddy currents to Gilbert damping should be negligible. Hence, to explain the thickness dependence of Gilbert damping the consideration of other mechanisms is necessary, and one of the possible mechanisms may be correlated with magnetoelastic properties. The correlation between the damping factor and the magnetoelastic properties has been already observed in Ni_x_Fe_1−x_ films^28^. However, a theoretical model describing quantitatively a correlation between Gilbert damping and magnetoelastic properties has not been developed so far. It was suggested^29^ that such model should take into account nonequilibrium statistical mechanics.

## Conclusions

The tetragonal distortion of the epitaxially grown Co_2_Fe_0.4_Mn_0.6_Si Heusler thin film, the magnetocrystalline anisotropy, anisotropic magnetoelastic properties as well as Gilbert damping all depend on both the thickness of the magnetic layer and the type of buffer layer on which the magnetic layer was deposited (Cr only or Cr covered by Ag).

The changes of a very large (of an order of 10^5^–10^6^ erg/cm^3^) perpendicular magnetocrystalline anisotropy qualitatively correlate with the changes of the in-plane tetragonal distortion. For most samples, however, this distortion is too small to explain the very large magnitudes of the perpendicular magnetocrystalline anisotropy, and to explain such large magnitudes, other mechanisms including both surface and volume effects must be also taken into account.

To describe the anisotropy of most samples, the in-plane component of the uniaxial anisotropy must be taken into account, and it is insufficient to consider only one perpendicular anisotropy constant.

It seems that for the CFMS layers of the thicknesses of an order of 50 nm or lower the influence of the eddy currents on the Gilbert damping factor is very small. Hence, to describe the changes in this damping factor with the magnetic layer thickness changes other mechanisms must be taken into account, and one of the possible mechanisms may be correlated with magnetoelastic properties. To explain the quantitative correlation between the Gilbert damping and magnetoelastic properties further investigations are necessary.

## Data Availability

All data reported in this manuscript is available from the corresponding author on a reasonable request.
